# Biocontrol Potential of Endophytic Actinobacteria against *Fusarium solani*, the Causal Agent of Sudden Decline Syndrome on Date Palm in the UAE

**DOI:** 10.3390/jof8010008

**Published:** 2021-12-23

**Authors:** Aisha A. Alblooshi, Gouthaman P. Purayil, Esam Eldin Saeed, Gaber A. Ramadan, Saeed Tariq, Amna S. Altaee, Khaled A. El-Tarabily, Synan F. AbuQamar

**Affiliations:** 1 Department of Biology, College of Science, United Arab Emirates University, Al Ain 15551, United Arab Emirates; 201110117@uaeu.ac.ae (A.A.A.); 202190196@uaeu.ac.ae (G.P.P.); jaber.ramadan@uaeu.ac.ae (G.A.R.); 201805992@uaeu.ac.ae (A.S.A.); 2 Khalifa Center for Genetic Engineering and Biotechnology, United Arab Emirates University, Al Ain 15551, United Arab Emirates; esameldin_saeed@uaeu.ac.ae (E.E.S.); 3 Department of Anatomy, College of Medicine and Health Sciences, United Arab Emirates University, Al Ain 15551, United Arab Emirates; stariq@uaeu.ac.ae (S.T.); 4 Harry Butler Institute, Murdoch University, Murdoch, WA 6150, Australia

**Keywords:** biocontrol, date palm, endophytic actinobacteria, *Fusarium solani*, sudden death syndrome, *Streptomyces*

## Abstract

Thirty-one endophytic streptomycete and non-streptomycete actinobacteria were isolated from healthy date palm root tissues. *In vitro* screening revealed that the antifungal action of isolate #16 was associated with the production of cell-wall degrading enzymes, whereas with diffusible antifungal metabolites in isolate #28, albeit their production of volatile antifungal compounds. According to the 16S rRNA gene sequencing, isolates #16 and #28 were identified as *Streptomyces polychromogenes* UAE2 (*Sp*; GenBank Accession #: OK560620) and *Streptomyces coeruleoprunus* UAE1 (*Sc*; OK560621), respectively. The two antagonists were recovered from root tissues until 12 weeks after inoculation, efficiently colonized root cortex and xylem vessels, indicating that the date palm roots are a suitable habitat for these endophytic isolates. At the end of the greenhouse experiments, the development of sudden decline syndrome (SDS) was markedly suppressed by 53% with the application of *Sp* and 86% with *Sc*, confirming their potential in disease management. Results showed that the estimated disease severity indices in diseased seedlings were significantly (*p* < 0.05) reduced from 4.75 (scale of 5) to 2.25 or 0.67 by either *Sp* or *Sc*, respectively. In addition, conidial numbers of the pathogen significantly (*p* < 0.05) dropped by 38% and 76% with *Sp* and *Sc*, respectively, compared to infected seedlings with *F. solani* (control). Thus, the suppression of disease symptoms was superior in seedlings pre-inoculated with *S. coeruleoprunus*, indicating that the diffusible antifungal metabolites were responsible for *F. solani* retardation in these plants. This is the first report of actinobacteria naturally existing in date palm tissues acting as microbial antagonists against SDS on date palm.

## 1. Introduction

Plant diseases caused by phytopathogens seriously result in the global crop yield reduction and severely cause billions of dollars’ worth of losses each year. Fungal diseases of plants can cause devastating damage to crops, threaten food security, and have the potential to hamper tourism [1,2,3]. At the present time, strategies of controlling plant fungal diseases are mainly dependent on the application of chemical fungicides [4], regardless of their negative association with the environmental pollution and human health hazards, and the induction of resistance to plant fungal pathogens. Safe, effective, and eco-friendly methods (i.e., biocontrol) may limit the presence of fungal pathogens and/or suppress their destroying activities [5,6,7]. Such methods have recently received more attention for management of fungal diseases.

Many species of microorganisms, such as fungi, non-filamentous bacteria, and actinobacteria, have been successfully used to control plant pathogens [6,7]. Actinobacteria are ubiquitous Gram-positive bacteria and are known for the production of various bioactive secondary metabolites [8]. About 45% of commercial metabolites are known to be produced by various actinobacteria [9,10]. Metabolites produced by some actinobacteria have antibacterial, antifungal, anticancer, antialgal, antimalarial, and anti-inflammatory activities that have been largely used in pharmaceutical and industrial fields [9,11].

In agriculture, actinobacteria can protect plants from a wide range of phytopathogenic fungi by the production of fungal cell-wall degrading enzymes (CWDEs), antifungal antibiotics [6,12,13,14,15], and siderophores [16]. Actinobacteria can also inhibit the growth of phytopathogenic fungi through hyperparasitism [17] and/or nutrient competition [18]. Actinobacteria can enhance the defensive capacity developed by a plant prior to infection/treatment that results in resistance against subsequent challenge by a pathogen, commonly known as induced systemic resistance (ISR) [19]. In addition, many genera of actinobacteria enhance plant growth and productivity by acting as plant growth promoters (PGPs) [20] through the production of plant growth regulators (PGRs) and/or the enzyme 1-aminocyclopropane-1-carboxylic acid (ACC) deaminase [14,15], and by increasing the availability of essential soil nutrients such as phosphorous [15,21].

Although species of *Streptomyces* (a genus of actinobacteria) are the most abundant microorganisms of the soil and rhizosphere microflora [22,23], they can also endophytically colonize various plant tissues [14,24]. Endophytic actinobacterial isolates possessing biological control activities effective against many pathogenic fungi have been identified [25,26,27]. They are also known for the production of secondary metabolites, including antibiotics, alkaloids, vitamins and CWDEs against their natural enemies. However, selection of these endophytic actinobacteria, mainly *Streptomyces*, can be determined according to their growth conditions and antifungal activities.

Date palm (*Phoenix dactylifera* L.) trees are important for heritage, cultural, religious, and economic reasons in the Arabian Peninsula [28]. In the United Arab Emirates (UAE), the commercial date production industry is solemnly threatened by black scorch disease and sudden decline syndrome (SDS) caused by the soil-borne fungi *Thielaviopsis punctulata* and *Fusarium* spp., respectively [1,29]. Particularly, *Fusarium solani* is considered as the most harmful fungal pathogen, attacking nearly all date palm cultivars, and thereby causing massive destruction of date palm plantations in the UAE [30]. Except for the chemical fungicide Cidely^®^ Top, there is no other effective chemical substance, physical agent, biological or integrated actions against *F. solani*. Taking into consideration the environmental pollution and human health, biocontrol is a prominent method to manage SDS and its causal agent on date palm.

In the current study, we aimed to identify endophytic actinobacteria with high antifungal activities to *F. solani* isolated from healthy root tissues of date palm plants, and to determine the effect of the metabolites secreted by the most promising isolates against *F. solani* under *in vitro* and greenhouse conditions.

## 2. Materials and Methods

### 2.1. Fungal Culture

The fungal pathogen, *F. solani* (DSM-106836), was previously identified as the causal agent of the SDS on date palm in the UAE [30]. *F. solani* was cultivated at 28 °C on potato dextrose agar (PDA; Lab M Limited, Lancashire, UK) plates (Sigma-Aldrich Chemie GmbH, Taufkirchen, Germany).

### 2.2. Isolation of Endophytic Actinobacteria from Date Palm Roots

Twelve free draining plastic pots (20 cm in diameter) were filled with four different soil samples collected from the rhizosphere of date palm trees growing in orchards located in four different campuses (Maqam, Falaj Haza, Islamic Institute and Al-Foah) of the United Arab Emirates University (UAEU) in Al-Ain city, UAE.

These soils were used to grow six-month-old healthy date palm seedlings (cv. Barhi) obtained from the Date Palm Development Research Unit-UAEU (DPDRU). The seedlings roots were surface-sterilized using 70% ethyl alcohol (EtOH) for 10 min and 5.25% commercial bleach for 10 min., followed by eight times washing with sterile distilled water. In each pot, two surface-sterilized date palm seedlings were planted for each soil sample and kept in a growth chamber under controlled environment conditions (16/8 h day/light at 30 °C and relative humidity (RH) of 60%).

After 8 weeks, roots growing in each soil sample were cut, pooled, and rinsed in running tap water, and the fresh root weight was recorded. Roots were surface-sterilized with propylene oxide (Sigma-Aldrich) vapor for 25 min [31], and 70% EtOH for 10 min and 5.25% commercial bleach for 10 min. This was followed by rinsing ten times in phosphate-buffered saline solution (PBS; pH 7.0), as recommended by [32].

To ensure the absence of microbial contaminants on the rhizoplane after surface disinfection, sterility checks were carried out. Briefly, 2–4 cm pieces of roots were plated out on tryptic soy agar plates (Lab M Ltd.) and PDA plates amended with 250 µg/mL chloramphenicol (Sigma-Aldrich). After 6 days of incubation in the dark at 28 °C, no bacterial or fungal growth indicated that the isolated strains were true endophytes.

In order to isolate endophytic actinobacteria from date palm root tissues, root samples were soaked in sterile PBS for 10 min, and macerated using Omni-mixer (Omni International, Kennesaw, GA, USA) for 20 min at 4000 rpm [33]. A series of dilutions (10^−2^–10^−5^) in PBS was carried out, and 200 µL aliquots were spread on inorganic salt starch agar (ISSA) amended with 50 μg/mL of nystatin and cycloheximide (Sigma-Aldrich) [34] using the spread plate technique. ISSA plates were dried for 10 min and then incubated at 28 °C in the dark for 10 days. For each root sample, three plates were used per dilution and the population densities were expressed as log_10_ colony forming units (cfu)/g fresh root weight [33].

All actinobacterial isolates were purified and transferred onto oatmeal agar plates supplemented with 0.1% yeast extract OMYEA (ISP medium 3) [34]. Preliminary identification of streptomycete actinobacteria (SA) and non-streptomycete actinobacteria (NSA) was based on morphological (stability of substrate mycelia, presence of aerial mycelia, and distribution of spores on the aerial and substrate mycelia) and cultural characteristics (color of aerial and substrate mycelia and production of diffusible pigment) [35]. Hyphae and/or spores of all actinobacterial strains were stored in 20% glycerol at −80 °C [36].

### 2.3. Screening of Biological Control Agents (BCAs) for the Production of CWDEs

All endophytic isolates obtained were evaluated for their potential to produce clear zones on *F. solani* mycelial fragment agar (MFA) as an indicator of preliminary production of CWDEs [37]. Plates were incubated in the dark at 28 °C for 7 days. Large diameters of clear zones (>30 mm) represented high CWDE activities. We also examined all obtained isolates for their abilities to secrete chitinase enzyme. Each isolate was inoculated onto colloidal chitin agar (CCA) plates and incubated in the dark at 28 °C for 7 days [38]. Colloidal chitin was prepared from crab shell chitin (Sigma-Aldrich) [39].

To detect the antagonistic isolates of highly active CWDEs and chitinase activities, large clear zone diameters (>30 mm) on both MFA and CCA plates, respectively, were selected. Six plates were used for each actinobacterial isolate.

### 2.4. Screening of BCAs for the Production of Diffusible Antifungal Metabolites

The cut-plug method [40] was used to evaluate the potential of all actinobacterial isolates to produce diffusible antifungal metabolites active against *F. solani*. The actinobacterial isolates were inoculated on fish meal extract agar (FMEA) plates [17] and incubated in the dark at 28 °C for 7 days. Plugs from *F. solani* culture were inoculated on PDA slants at 28 °C until sporulation, which were then flooded with 50 mM phosphate buffer, pH 6.8 [5]. Spores and mycelial fragments were homogenized using Omni-mixer (Omni International) for 20 min at 4000 rpm. The obtained supernatants were aseptically added to PDA bottles (50 °C), mixed, and poured into petri plates. The *F. solani* inoculum was adjusted to ~10^8^ conidia/mL.

Plugs were transferred from the actinobacterial cultures on FMEA with a sterilized 11 mm cork-borer onto PDA plates seeded with *F. solani*. Plates were incubated in the dark at 28 °C for 5 days and the diameters of inhibition zones were determined. The most successful antifungal metabolite-producing isolates exhibiting the largest zone of inhibition (>30 mm) were selected for further experiments, and the remaining of isolates were not used in the subsequent experiments. PDA plates with non-inoculated agar plugs served as control. Six plates were used for each actinobacterial isolate.

### 2.5. Bioassays for the Production of Volatile Antifungal Compounds, Siderophores, and Hydrogen Cyanide (HCN)

The promising BCAs from experiments 2.3 and 2.4 were also tested on FMEA for the production of volatile antifungal compounds [41]. FMEA plates were inoculated with the antagonists by evenly spreading spores and/or mycelial fragments from a 7-day-old culture of each isolate on the entire surface of the FMEA plate. These cultures were incubated in the dark for 10 days at 28 °C. The plates were then inoculated with an actively growing *F. solani* mycelial plug (5 mm diameter). The plate lids were removed and the plates with the pathogen were inverted over the actinobacteria plates. Two layers of Parafilm (American National Can TM, Greenwich, CT, USA) were used to tape the two plate bases together. Control plates were similarly prepared except that a non-inoculated plate was used instead of a plate containing the antagonist. All plates were incubated for 7 days at 28 °C in the dark. Six plates were used for each actinobacterial isolate. The colony diameter of *F. solani* was measured and compared to that of the control after 7 days.

Crome azurol S (CAS) agar plates [42] was used to test the abilities of the actinobacterial isolates to produce siderophores. CAS agar plates were inoculated with each BCA and incubated in the dark for 7 days at 28 °C. The formation of a yellow–orange halo zone around and beneath the colonies was considered positive for the production of siderophores [43]. Six plates were used for each actinobacterial isolate.

Plates of tryptic soy agar medium (Lab M Limited) supplemented with 4.4 g/L glycine were used to evaluate the isolates for their abilities to produce HCN. These plates were inoculated with each BCA and consequently inverted. A piece of filter paper soaked in 2% sodium carbonate and 0.5% picric acid was placed in the lid of each petri dish, and incubated at 28 °C [44]. The discoloration of the filter paper to orange–brown indicates the production of HCN [45]. Six plates were used for each actinobacterial isolate.

### 2.6. Determination of the Population Densities of Endophytic Actinobacteria Isolated from Date Palm

Three antagonistic isolates (#5, #16, and #17) out of seven isolates from experiment 2.3 that showed the strongest production of CWDEs and three antagonistic isolates (#22, #28, and #29) out of nine isolates from experiment 2.4 that showed the strongest production of diffusible antifungal metabolites were selected to test their abilities to colonize the internal root tissues of date palm and to study their population densities and their persistence for a period of 12 weeks.

Rifampicin-resistant mutants of the promising antagonistic six isolates were selected on ISSA medium amended with 100 µg/mL rifampicin (Sigma-Aldrich) and tested according to [46]. These mutants were also compared to the corresponding wild type strains for the production of diffusible antifungal metabolites and CWDEs. Morphological features, colony morphology, CWDEs, and antifungal metabolites production of mutants were found to be similar to those of wild type strains.

To prepare the BCA inoculum for *in vivo* experiments in the greenhouse, 4 mL aliquots of 20% glycerol suspension of the six endophytes were individually inoculated into 250-mL inorganic salt starch broth (ISSB) and shaken on orbital shaker (New Brunswick Scientific, Edison, NJ, USA) for 5 days at 250 rpm. Mycelium and/or spores were centrifuged (12,000× *g*) for 15 min at 20 °C, and the pellet was suspended in 10 mL PBS and re-centrifuged [47]. For each suspension, 0.1 mL of each 10^−3^, 10^−4^, 10^−5^, and 10^−6^ dilutions was made in PBS and spread over ISSA plates which were incubated for 5 days at 28 °C. The final concentration of each isolate was adjusted to be ~10^8^ cfu/mL and was used as an inoculum.

Six-month-old date palm seedlings were inoculated with individual isolates using the pruned-root dip method [48] in order to assess colonization of internal root tissues. Briefly, date palm roots were trimmed to uptake the actinobacterial inoculum. Seedlings were kept in sterile containers for 6 h at 25 °C with their roots immersed in the inoculum suspension of each isolate (10^8^ cfu/mL). Control seedlings were treated with non-inoculated ISSB. All seedlings were planted in free draining pots, filled with 8 kg of soils collected from Al-Foah Farm described in Section 2.2. Pots were placed in an evaporative-cooled greenhouse (25 ± 2 °C, relative humidity of 60 ± 5% and photosynthetic photon flux density of 700 µmol/m^2^ s). Seedlings were watered daily to container capacity and each treatment was replicated eight times and each replicate was determined by a single pot containing one seedling. After planting, roots were sampled weekly (for 12 weeks), thoroughly washed with water, and surface-sterilized as described in Section 2.2.

Root tissues form date palm seedlings previously inoculated with individual six isolates were homogenized as previously described [33]. The slurry was filtered through sterile cotton cloth and the filtrate was serially diluted (10^−2^, 10^−3^ and 10^−4^). Aliquots (0.2 mL) were spread on ISSA plates supplemented with rifampicin, cycloheximide and nystatin (Sigma-Aldrich). For each root sample dilution, six replicated plates were dried in a laminar flow-cabinet for 15 min and the plates were incubated in the dark for 7 days at 28 °C [47]. The population density (PD) of endophytic actinobacteria was calculated as log_10_ cfu/g fresh root weight [33].

### 2.7. Assays of Antifungal Metabolites and CWDEs Activities

Based on the results from the internal root colonization and population densities experiment (Section 2.6), only the most promising antagonistic BCAs (isolate #16 and #28) producing the strongest inhibition of *F. solani*, and which showed the strongest root colonization and the highest population densities up to 12 weeks, were selected for all the experiments described below. The remaining isolates were not considered in the subsequent studies because they either produced no or very low levels of inhibition against the fungus and were not efficiently active in the colonization of internal roots.

These two isolates were also examined for their abilities to produce diffusible antifungal metabolites active against *F. solani* using the cup plate technique [49]. Individual 250 mL Erlenmeyer flasks containing 50 mL of sterile fish meal extract broth (FMEB) [17] were inoculated with 1 mL of 10% glycerol suspension of the BCA (approximately 10^8^ cfu/mL) and incubated in an orbital shaker (New Brunswick Scientific) in the dark at 28 °C at 200 rpm. After 7 days of incubation, the suspensions from each flask were centrifuged at 12,000× *g* for 30 min. The crude culture filtrate (supernatant) was filtered through sterile 0.22 µm pore size Millipore membranes (Millipore Corporation, Billerica, MA, USA). The crude culture filtrate was kept in sterilized tubes and stored at 4 °C.

Inocula for the preparation of the *F. solani* seeded PDA plates were prepared as described above for the cut-plug technique. Wells were cut in the centers of the fresh PDA plates seeded with *F. solani* using a sterilized 11-mm cork-borer. Aliquots (0.3 mL) of the filter-sterilized crude culture filtrate were introduced into the wells using a sterilized syringe. The plates were incubated for 5 days in the dark at 28 °C; and the diameters of zones of inhibition were measured in mm. Filter-sterilized ISSB without the BCA (as a control) was similarly added into the wells in the PDA plates seeded with *F. solani*. Six plates were used for each actinobacterial isolate.

To further assay inhibition of *F. solani* by the BCA, we conducted a dialysis membrane overlay technique on FMEA [5,50]. Dialysis membrane (90-mm in diameter; Type 45311; Union Carbide Corporation, Alsip, IL, USA) was overlaid on FMEA or CCA. The surface of the membrane was inoculated with the BCA by spreading mycelial fragments and/or spores of a 7-day-old culture of the BCA cultivated on OMYEA. The plates were incubated at 28 °C in the dark. The membranes with the adhering cultures were removed from the agar plates after 10 days of incubation. The center of each plate was inoculated with a disc (5-mm in diameter) of *F. solani* culture grown on FMEA at 28 °C in the dark. After 8 days, colony diameters of *F. solani* were measured (mm) and compared to that of FMEA control plates where the pathogen was grown without the addition of the BCA. At the end of the incubation period, agar plugs showing no pathogen growth were further transferred to a fresh PDA plate and incubated at 28 °C for 5 days. The purpose of this step was to test if the metabolites diffusing through the dialysis membrane were fungistatic (*F. solani* growth from the plug) or fungicidal (no *F. solani* growth from the plug).

To quantitatively determine the concentration of chitinase and ß-1,3-glucanase produced by the two BCAs, the minimal synthetic medium supplemented with 2 mg/mL of either colloidal chitin or laminarin (Sigma-Aldrich), respectively, was used [51,52]. The specific activity of chitinase- or ß-1,3-glucanase was calculated by the release of N-acetyl-D-glucosamine from colloidal chitin or the amount of reducing sugars liberated from laminarin using dinitrosalicylic acid solution, respectively [53,54]. Folin phenol reagent method [55] was used to determine the protein content of the enzyme solution.

### 2.8. Evaluation of Crude Culture Filtrates of BCA on F. solani

*In vitro* assays for inhibition of colony and mycelial growth were conducted on PDA plates [56]. BCA #16 and #28 were inoculated on FMEB or colloidal chitin broth (CCB) [38] for 7 days at 28 °C.

CCB or FMEB were inoculated with 2 mL of a 20% glycerol suspension of BCA #16 and #28 (10^8^ cfu/mL), respectively. The flasks were kept on an orbital shaker (New Brunswick Scientific) (250 rpm) at 28 °C. After 7 days, flasks were centrifuged at 12,000× *g* for 30 min at 4 °C. The supernatant was then filtered using 0.22 µm Millipore membranes (Millipore Corporation).

The crude culture filtrate (either from FMEB for isolate #28 or CCB for isolate #16) was mixed with sterilized PDA at 0% (control), 25%, 50%, and 100% proportions and poured into petri plates. The medium was inoculated with an agar plug (5-mm in diameter) of *F. solani*. After 5 days of incubation in the dark at 28 °C, the colony diameter of *F. solani* was compared with that of the control. Six plates were used for each actinobacterial isolate.

Inhibition of mycelial growth was also conducted in potato dextrose broth (PDB) [56]. Prepared crude culture filtrates (either from FMEB for isolate #28 or CCB for isolate #16) were mixed with sterilized PDB at 0%, 25%, 50% and 100% proportions. The PDB was inoculated with a 5-mm diameter agar plug of *F. solani* mycelium. After 10 days of incubation in the dark at 28 °C, the mycelial dry weight (g) of *F. solani* was measured. Six flasks were used for each actinobacterial isolate.

To determine the effect on conidia germination and germ tube elongation of *F. solani*, crude culture filtrate of the BCAs (either from CCB for isolate #16 or FMEB for isolate #28) was prepared according to [56]. Aliquots (20 µL) of the crude culture filtrate were mixed with 20 µL of conidial suspension of *F. solani* and 60 µL of PDB (control consisted of no BCA). The reaction mixture was incubated at 28 °C in the dark. After 24 h, the percent of spore germination and average length of germ tubes (µm) were microscopically determined at 400X using a light microscope (Nikon-Eclipse 50i; Nikon Instruments Inc., Melville, NY, USA) and compared with the control.

We also studied the effect of the crude culture filtrate of the two BCAs on hyphal morphology. *F. solani* was grown in 100-mL PDB for 10 days at 28 °C in the dark. After the removal of the culture broth, mycelial mats were aseptically washed four times with sterile distilled water [57]. Carbon-deficient salt solution (100 mL) and crude culture filtrate of the BCA (50 mL) (either from FMEB for isolate #28 or CCB for isolate #16) were added to the mycelial suspension of *F. solani*. Flasks were incubated at 28 °C in the dark for 4 days. Carbon-deficient salt solution containing *F. solani* mycelium without BCAs served as a control. Any subsequent changes in the hyphal morphology of *F. solani* were observed at 1000X using a LM (Nikon-Eclipse 50i) connected with Nikon camera (DS—Flic). Six replicates were used for each sampling.

### 2.9. Identification of the BCAs

The BCAs (isolate #16 and #28) were identified to the species level based on their cultural, morphological, biochemical, and molecular characteristics [58]. Nikon-Eclipse 50i LM and Philips XL-30 SEM (FEI Co., Eindhoven, The Netherlands) were used for the light microscope (LM) and scanning electron microscopy (SEM), respectively. In addition, both isolates were further confirmed by 16S rRNA gene sequence analysis done by the Deutsche Sammlung von Mikroorganismen und Zellkulturen GmbH, (DSMZ), Braunschweig, Germany. The partial 16S rRNA gene sequence (1487 and 1519 bp in isolate #16 and #28, respectively) was also determined by direct sequencing of PCR-amplified 16*S* rRNA [59].

PCR conditions were adjusted according to [5]. Phylogenetic tree was constructed to predict the species level of the studied isolate using the neighbor-joining method implemented in Molecular Evolutionary Genetics Analysis 7.0 (MEGA7) software [60,61].

### 2.10. Light and Transmission Electron Microscopy

For LM and transmission electron microscopy (TEM), date palm root samples inoculated with isolate #16 or #28 were fixed with karnovsky’s fixative in 0.17 M phosphate buffer at pH 7.2 containing 2% paraformaldehyde (Sigma-Aldrich) and 2.5% glutaraldehyde (Sigma-Aldrich) for 24 h at 4 °C. Tissues were rinsed three times in 0.17 M phosphate buffer (pH 7.2) and post-fixed with 1% aqueous osmium tetroxide for 2 h at 25 °C. Tissues were dehydrated with a series of ascending grades of EtOH (30%–100%) and soaked into the propylene oxide (Sigma-Aldrich). Finally, tissue samples were infiltrated and embedded in epoxy resin (Epon 812, Agar Scientific, Essex, UK) and polymerized at 60 °C in embedding oven for 24 h [62].

Tissues blocks were trimmed, where semi-thin sections (1.5 µm) and ultra-thin sections (95-nm) were cut with ultramicrotome Leica EM UC7 (Leica Microsystems, Vienna, Austria). Heat-dried semi-thin sections of root specimen were stained with a mixture of 1% toluidine blue and borax (Sigma-Aldrich). For LM analysis, slides were observed using Olympus BH2 (Olympus Optical Co., Ltd., Nagano, Japan) LM equipped with an LM Digital Camera and Software (Jenoptik ProgRes Camera, C12plus, Jena, Germany).

Ultra-thin sections (90-nm) were then collected on 200 mesh copper grids and contrasted with 10% uranyl acetate, followed by 3% lead citrate. Finally, the grids were examined under Tecnai Spirit G2 Biotwin TEM operating at 80 kV (FEI Co., Indore, The Netherlands).

### 2.11. Disease Assays, Spore Counts, and Disease Severity Index (DSI) in the Greenhouse

In the nursery experiment, the strongest BCAs were evaluated on date palm seedlings. Our aim was to test the efficacy of BCAs prior infection with *F. solani* on date palm. Except for the extended inoculation time point using the individual BCAs, pathogenicity tests and inoculation methods with the pathogen and BCA were described in Section 2.6. Seedlings were treated with each BCA for 5 days before *F. solani* infection to ensure root colonization by that particular BCA.

For disease assays in the greenhouse experiments, roots of six-month-old date palm (cv. Barhi) seedlings, obtained from DPDRU-UAEU, were mechanically wounded with sterilized scalpels prior to inoculation. Seedlings were inoculated with 10 mL of inoculum (14-day-old Miracloth-filtered fungal cultures) on roots that were separately dipped for 1 h in the inoculum suspension of *F. solani*. The conidial concentration was determined using hemocytometer (Agar Scientific Limited, Essex, UK) and adjusted to 10^6^ conidia/mL [30].

Control plants were dipped in 10 mL of sterile distilled water. The inoculated seedlings were covered with transparent polyethylene sheet for 72 h. Seedlings in free draining pots (20-cm in diameter), filled with 8 kg of soils collected from Al-Foah Farm described in Section 2.2 were randomly grown in an evaporative-cooled greenhouse. Seedlings were watered daily to container capacity and examined for disease development.

To evaluate the effectiveness of BCA treatments on date palm plants. The treatments used in this experiment were as the following:

(i). Healthy controls (C): Non-inoculated seedlings with any of the BCAs or *F. solani*;
(ii). Diseased controls (*Fs*): Inoculated-seedlings with *F. solani* only;
(ii). BCA controls (*Sp* or *Sc*): Inoculated-seedlings with either *S. polychromogenes* UAE2 (isolate #16) (BCA1) or *S. coeruleoprunus* UAE1 (isolate #28) (BCA2) only; and
(iv). *Sp* + *Fs* or *Sc* + *Fs*: Inoculated-seedlings with *S. polychromogenes* UAE2 (isolate #16) (BCA1) or *S. coeruleoprunus* UAE1 (isolate #28) (BCA2) at 5 days before *F. solani* inoculation.

Date palm seedlings were inoculated with the endophytic isolates using the pruned-root dip method as described in Section 2.6. Eight plants in separate pots, arranged in a completely randomized design on a bench in the greenhouse, were used for each treatment/group. Plants were inoculated with *F. solani* and grown for additional 10 and 30 days (days post inoculation; dpi) corresponding to 15- and 35-days post treatment (dpt), respectively. The experiment was independently conducted and repeated two times.

At 35 dpt, the fungal conidia of known weight of affected tissues from inoculated plants (*n* = 8) cut into small pieces (2–5 mm in diameter) were macerated in 5-mL of distilled water and vigorously shaken for 30 min. For each treatment, the harvested conidia were counted using hemocytometer [2,30].

For all inoculated seedlings, DSI was recorded for SDS symptoms at 35 dpt using a scale of 0–5: 0 = no apparent symptoms, 1 = 1–10% necrotic or white area in leaves or rotting in roots, 2 = 11–25%, 3 = 26–50%, 4 = 51–75%, and 5 = 76–100% [30,63]. All experiments were independently repeated twice with similar results.

### 2.12. Statistical Analyses

For *in vitro* evaluation of BCAs against *F. solani*, data were analyzed using the analysis of variance (ANOVA). Means were separated using Duncan’s multiple range test at 5% level of significance. These experiments were repeated in triplicates using six plates/treatment for each time with similar results.

Fungal spore counts and DSI of the *in vivo* treatments against *F. solani* were analyzed using ANOVA and Duncan’s multiple range test to determine the statistical significance at *p* < 0.05. For each experiment, data represent mean ± standard error (SE) from eight plants. These experiments were independently repeated twice, and similar results were obtained in all replicates. Statistical analyses were performed using SAS Software version 9 (SAS Institute Inc., Cary, NC, USA).

## 3. Results

### 3.1. In vitro Screening of Endophytic Actinobacteria for CWDEs Activities and Antibiosis on F. solani

A total of 31 different endophytic actinobacteria were isolated according to their cultural characteristics on ISSA plates, of which 24 SA (77.4%) and 7 NSA (22.6%) isolates were identified (Figure S1). Isolates of *Actinoplanes, Dactylosporangium*, *Micromonospora*, and *Microbispora* were identified as the major species of NSA (Table 1).

First, we *in vitro* tested the isolates according to their antagonism against *F. solani*. Five SA (#5, #13, #16, #24, and #31) and two NSA (#17 and #21) were ranked as highly active CWDEs. These seven isolates produced large clearing zones (>30 mm) around the colony on both CCA (Figure S2) and *F. solani* MFA (Table 1; Figure S2) plates; thus, showing significant (*p* < 0.05) difference in their antagonism. The remainder of isolates produced small clearing zones of <30 mm and were eliminated from further assessment.

By using the cut-plug (Table 1; Figure S2) and cup-plate techniques (Figure S2), nine isolates, representing 35.5% of all endophytic actinobacteria obtained, were able to produce strong diffusible antifungal metabolites against *F. solani*. Six SA (#1, #7, #15, #22, #25, and #28) and three NSA (#3, #8, and #29) produced large zones of inhibition against *F. solani* in these experiments, ranging between 31.4 mm in isolate #22 and 47.8 mm in isolate #29 (Table 1). The remaining isolates were not considered because they produced no or negligible levels of inhibition (<30 mm) against *F. solani*. This suggests that the isolated endophytic SA and NSA from the date palm root samples had antifungal activities against the fungal pathogen *F. solani*. Apparently, none of the 17 tested isolates were, however, able to produce both diffusible antifungal metabolites and CWDEs (Table 1).

The production of volatile antifungal compounds and siderophores varied among the 16 isolates tested (Table 1). Isolates #7, 8, #9, #13, #24, #25, and #31 failed to produce volatile antifungal compounds; whereas isolates #1, #3, #9, #15, #21, and #24 failed to produce siderophores (Table 1). Interestingly, four SA isolates (#5, #16, #22, and #28) and two NSA belonging to *Micromonospora* species (#17 and #29) had the ability to produce both volatile antifungal compounds and siderophores.

Although CWDEs-producing (#5, #13, #16, #17, and #31) and diffusible metabolites-producing (#7, #8, #22, #25, #28, and #29) isolates produced siderophores (Table 1; Figure S3), none of the isolates were considered an HCN producer (Table 1). Altogether, our *in vitro* data suggest that the most promising actinobacterial isolates produced volatile compounds and siderophores, in addition to their antagonistic effect to *F. solani* through CWDEs (#5, #16 and #17) and antibiosis (#22, #28 and #29). These isolates were selected for further analyses and considered as BCA candidates.

### 3.2. Total Population of Endophytic Candidates of BCA in Date Palm Roots

**Figure 1:**
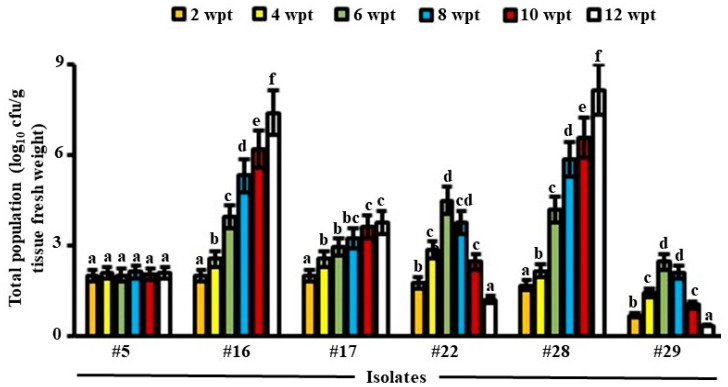
Total population of potential BCAs in date palm roots. The tested endophytic actinobacterial populations in root tissues of date palm grown under greenhouse conditions were sampled at different time points. Values are means of eight replicates ± SE for each sampling from two independent experiments. Mean values followed by different letters are significantly (*p* < 0.05) different according to Duncan’s multiple range test. Isolates #16 and #28 represent *Streptomyces polychromogenes* UAE2 (BCA1) and *Streptomyces coeruleoprunus* UAE1 (BCA2), respectively. BCA, biological control agent; wpt, weeks post treatment.

In general, the promising three isolates producing CWDEs (#5, #16 and #17) or the three isolates possessing antibiosis (#22, #28 and #29) were recovered from internal root tissues of date palm at all sampling time points (up to 12 weeks), but with different levels of colonization, thus showing their endophytic nature and sustaining healthy plants. Except for #5, the total population of isolates increased significantly (*p* < 0.05) in all examined weeks in root tissues (Figure 1). Based on our results, colonization of isolate #17 inside roots was limited. This was evident from the insignificant (*p* > 0.05) mean of total population in this isolate between weeks 4–8 and 10–12 in roots of date palm seedlings (Figure 1).

We also noticed a significant (*p* < 0.05) increase until week 6 inside root tissues, followed by a reduction in PD of isolate #22 starting week 8 until the end of the experiment. A similar pattern of PD inside root tissues of date palm was found for isolate #29. This indicates that isolate #22 and #29 did not sufficiently recover from tissues in week 6 onward. This suggests that these isolates did not colonize internal tissues efficiently. Therefore, isolates #5, #17, #22, and #29 were not included in the subsequent greenhouse trials.

For the CWDEs-producing isolate #16, PD dramatically increased for the tested 12-week colonization in root tissues (Figure 1), thus suggesting beneficial plant-microbe interactions i.e., BCA. From the beginning until the end of the colonization period, similar increase patterns in PD within the roots for isolate #28 possessing antibiosis properties were observed, anticipating it as a perfect BCA for the greenhouse experiments. Therefore, only isolates #16 and #28 were chosen as ideal BCAs to suppress SDS in date palm.

**Figure 2:**
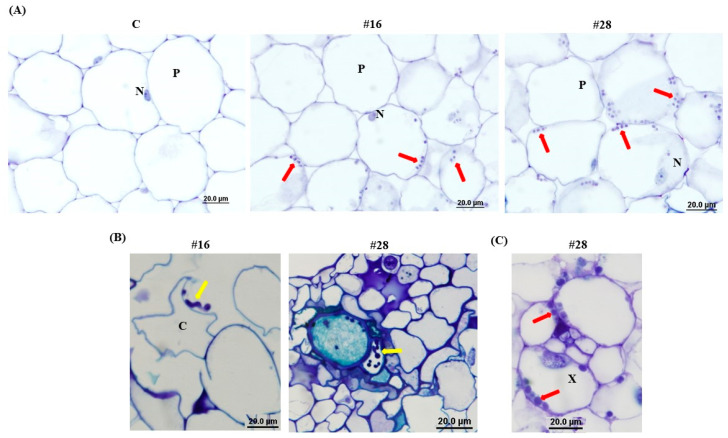
Colonization of endophytic BCAs in root tissues of date palm. Light micrograph of semi-thin sections of date palm root tissues (**A**) non-inoculated control (C; left), or inoculated with either isolate #16 (BCA1; middle) or #28 (BCA2; right) (1000X); (**B**) spore germination within the root cortex cells inoculated with either isolate #16 (left) or #28 (right) (1000X) and (**C**) spores of isolate #28 in the root xylem of date palm (1000X). In (**A**–**C**), all sections were stained with 0.1% toluidine blue showing the distribution of spores in root tissues (red arrows) and germinating spores within the root cells (yellow arrows) of date palm. Bars: 20 µm. Isolates #16 and #28 represent *Streptomyces polychromogenes* UAE2 and *Streptomyces coeruleoprunus* UAE1, respectively. BCA, biological control agent; P, pith, N, nucleus; C, cortex; X, xylem.

Tissue-associated with actinobacterial isolates #16 and #28 were also examined using LM. After 5 weeks of inoculation, spores of both isolates were abundantly present within date palm-inoculated roots (Figure 2A). Mycelial growth carrying spore chains, as well as germinating spores of both isolates within cortical and xylem cells of roots, were also detected (Figure 2B,C).

**Figure 3:**
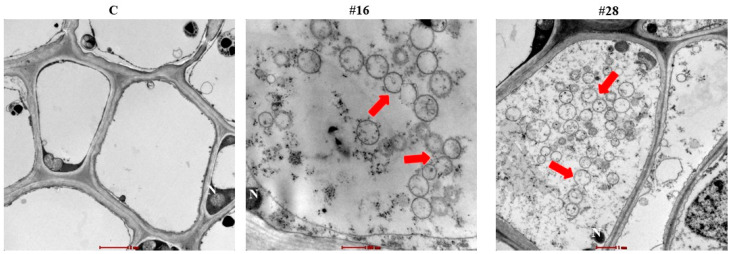
Intercellular colonization of date palm root tissues by the endophytic BCAs. Transmission electron micrograph of ultra-thin sections of date palm root tissues of non-inoculated control (C; left; 6000X) and inoculated with isolate #16 (BCA1; middle; 20,500X) or #28 (BCA2; right; 11,500X). All sections were stained with uranyl acetate and lead citrate showing the distribution of spore chains (red arrows). Bars: 2 µm (**left**); 500 nm (**middle**); 1 µm (**right**). Isolates #16 and #28 represent *Streptomyces polychromogenes* UAE2 and *Streptomyces coeruleoprunus* UAE1, respectively. BCA, biological control agent; N, nucleus.

Ultra-thin sections of inoculated seedlings with isolates #16 and #28 revealed that they could intercellularly colonize within cortical cells and xylem vessels of the root tissues (Figure 3). Altogether, our data confirmed the endophytic nature of isolates #16 and #28 by successfully residing within root tissues of date palm.

### 3.3. In vitro Evaluation of Antagonistic Properties of the BCA

The filter-sterilized crude culture filtrate obtained from FMEB of isolate #28 (BCA2), introduced into the wells using the cup plate technique, caused significant (*p* < 0.05) retardation of the growth of *F. solani* with an inhibition zone of 58.4 mm, when compared to the antifungal metabolite non-producing isolate #16 (BCA1) or control (Table S1; Figure S2). In contrast to isolate #28 or control, the diffused antifungal metabolites of isolate #16 on CCA plates inhibited the growth of *F. solani* inoculum after removing the dialysis membranes (Table S1; Figure S3). The pathogen did not recover from the plugs when transferred from treated plates to fresh PDA. This indicated that BCA1 showed fungicidal activities to *F. solani* through the secretion of CWDEs. On other hand, the growth of *F. solani* was clearly inhibited by the diffused metabolites of BCA2 only after removing the dialysis membranes from the FMEA, compared to control or BCA1 (Table S1; Figure S3). In addition, the pathogen failed to grow from the plugs transferred from the treatment plates to fresh PDA in the absence of diffused metabolites, confirming that the antifungal diffusible metabolites of BCA2 were clearly fungicidal to *F. solani*.

Chitinase production by BCA1 was significantly (*p* < 0.05) higher in the media amended with colloidal chitin than on media amended with *F. solani* cell walls (Table S1). BCA1 could also produce ß-1,3-glucanase when grown on media amended with laminarin or *F. solani* cell walls. The production of ß-1,3-glucanase was found to be significantly (*p* < 0.05) higher on laminarin-amended medium. On the other hand, there were no detectable levels of chitinase or ß-1,3-glucanase by BCA2 when it grew in media containing either colloidal chitin or *F. solani* cell walls, or in media containing laminarin or *F. solani* cell walls, respectively (Table S1).

### 3.4. Effect of BCA Candidates on Hyphal and Cytoplasmic Integrity of F. solani

When compared with the control, the crude culture filtrates of isolate #16 (BCA1) from CCB dramatically inhibited colony growth on PDA plates and mycelial dry weight of *F. solani* on PDB, with the increasing levels of crude culture filtrates after 5 days of incubation at 28 °C (Table 2). Isolate #16 significantly (*p* < 0.05) decreased colony diameter of *F. solani* by 42.4% and 84.1% when its culture filtrate increased from 0% to 25% and from 0% to 50%, respectively. About 40.4% and 81.9% of the mycelial dry weight of *F. solani* was also reduced when 25% and 50% culture filtrate of isolate #16 was applied, respectively. Filter-sterilized crude culture filtrates of isolate #28 (BCA2) from FMEB were effective in inhibiting growth of *F. solani* by 36.8% (from 0% to 25%) and 86.5% (from 0% to 50%) (Table 2). In addition, the crude culture filtrates of BCA2 from FMEB significantly (*p* < 0.05) decreased the mycelial dry weight of the pathogen when proportionally added into PDB. When culture filtrate of isolate #16 from CCB or isolate #28 from FMEB reached 100%, we did not observe any colony or mycelial growth of *F. solnai* (Table 2).

Similarly, a significant (*p* < 0.05) reduction was observed in the germination of conidia and the average length of germ tubes produced by *F. solani* when exposed to the crude culture filtrate of BCA1 (in CCB) and BCA2 (in FMEB) after 24 h of incubation (Table 2). Although >85% of *F. solani* conidia had germinated when no BCA1 or BCA2 was added, only ~1% germinated when 100% culture filtrate of either BCA1 or BCA2 was applied. In addition, the more culture filtrate of BCA1 or BCA2 supplied, the shorter germ tube of *F. solani* obtained. This indicates that the major biodeterioration occurred on *F. solani* by BCA1 and BCA2.

**Figure 4:**
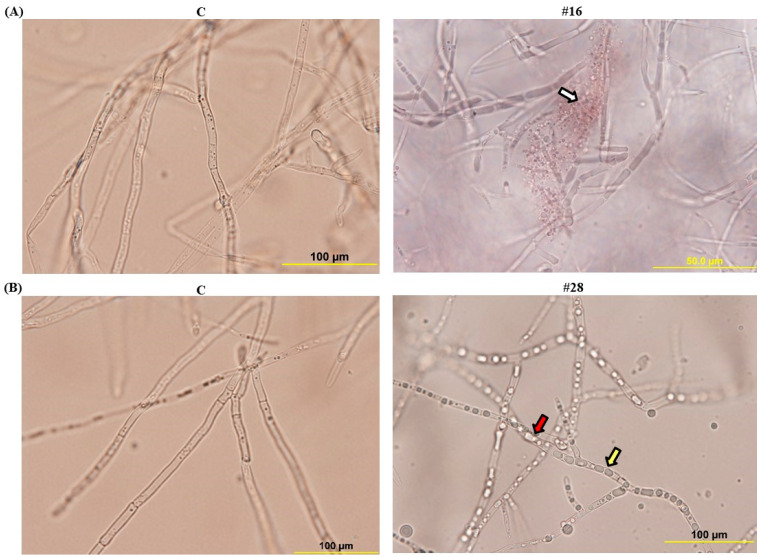
Effect of the BCAs on hyphae and cytoplasm of *Fusarium solani*. Abnormalities observed in the hyphal morphology including septum formation and cytoplasmic contents of *F. solani*, following exposure to (**A**) isolate #16 on CCB; or (**B**) filter-sterilized crude culture filtrate of isolate #28 on FMEB, compared to their corresponding control (C). White arrow indicates cytoplasmic lysis. Yellow and red arrows indicate hyphal non-septate formation and cytoplasmic coagulation, respectively. Light microscopy images were taken at 1000X magnification. Isolates #16 and #28 represent *Streptomyces polychromogenes* UAE2 (BCA1) and *Streptomyces coeruleoprunus* UAE1 (BCA2), respectively. BCA, biological control agent; CCB, colloidal chitin broth; FMEB, fish meal extract broth.

We also determined the effect of the crude culture filtrate of BCA1 and BCA2 obtained from CCB and FMEB, respectively, on hyphal growth of *F. solani* (Figure 4). All mycelial components in control flasks were not affected and seemed intact. The fungal pathogen treated with the crude culture filtrate of BCA1 obtained from CCB, showed hyphal lysis (Figure 4A). However, BCA2 caused hyphal abnormalities and cytoplasmic coagulation of *F. solani* after the treatment with the crude culture filtrate of BCA2 on FMEB (Figure 4B). This confirms that the two BCA candidates have antifungal activities against *F. solani*, albeit their different mode of actions.

### 3.5. Identification of the BCA Candidates to the Species Level

The potential antagonistic BCA candidates were identified using 16S rRNA gene sequencing. The resulting sequence data from isolate #16 and #28 were deposited in NCBI (GenBank Accession #: OK560620 and OK560621, respectively). In addition, a comparison with representative 16S rRNA gene sequences of organisms belonging to actinobacteria was carried out using phylogenetic analysis.

Comparison of the 16S rRNA gene of isolate #16 (BCA1; ∼1486 bp) with sequences in the GenBank database revealed that the BCA candidate was a streptomycete sp. with 99.8% similarity to *Streptomyces polychromogenes* NBRC 13072 (NR041109) and 99.7% to *Streptomyces* *racemochromogenes* NRRL B-5430 (NR043499; Figure S4). The rest of the *Streptomyces* spp. showed less than 98.7% similarity with the target antagonistic strain. To obtain a more definitive identification of the isolate, pure cultures of isolate #16 were cultivated on ISP medium 3. After 14 days of cultivation, the cultures of the isolate were grayish to pinkish colored-aerial mycelium and yellowish-brown substrate mycelium (Figure S4). Using SEM, the configuration of the spore chains displayed long, straight spore chain of >50 spores per chain, which belongs to section rectiflexibiles (Figure S4). In general, spore chains were long chains of spores, and the spore surface was smooth. Knots and nest-like tangles were also seen. Together, this suggests that the outstanding isolate #16 (BCA1) can be identified as *Streptomyces polychromogenes* (Hagemann et al. 1964) [64] Strain UAE2.

**Figure 5:**
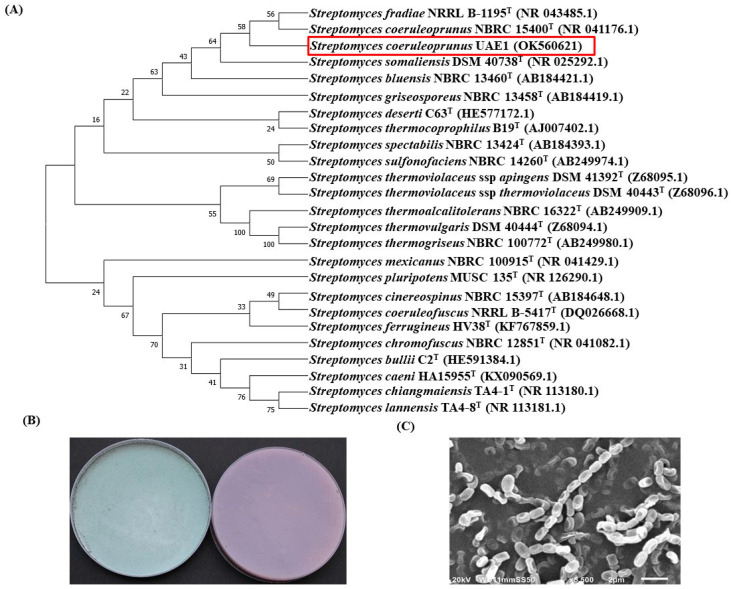
Molecular, cultural, and morphological characteristics of *Streptomyces coeruleoprunus* UAE1. Identification of *S. coeruleoprunus* UAE1 (BCA2) according to (**A**) the dendrogram that showing the phylogenetic relationships between *S. coeruleoprunus* UAE1 (NCBI accession #: OK560621) and other members of *Streptomyces* spp. based on the 16S rRNA sequences prepared by the neighbor-joining method; (**B**) light blue aerial mycelium (left) and pink to gray-violet substrate mycelium (right) growing on (ISP medium 3) supplemented with yeast extract, and (**C**) scanning electron micrograph (5500X) of the straight spore chains (section rectiflexibiles) and smooth-surfaced spores of *S. coeruleoprunus* UAE1. In (**A**), numbers at nodes indicate percentage levels of bootstrap support based on a neighbor-joining analysis of 500 resampled datasets. GenBank accession numbers are given in parentheses. BCA, biological control agent.

Phylogenetic analysis of 16S rRNA of BCA2 (isolate #28) showed 99.5% similarity to *Streptomyces* *coeruleoprunus* NBRC 15400 (NR 041176) and *Streptomyces* *fradiae* NRRL B-1195 (NR 043485; Figure 5A), while the other *Streptomyces* spp. showed <98.6% similarity. Typical light blue aerial mycelia and pink to gray-violet substrate mycelia of BCA2 culture were observed (Figure 5B). This isolate showed straight spore chains (section rectiflexibiles) and smooth-surfaced spores (Figure 5C). This suggests that BCA2 (#28) could be most probably recognized as *Streptomyces* *coeruleoprunus* (Preobrazhenskaya 1986) [65] strain UAE1.

### 3.6. Anatgonism of the Tested Streptomyces spp. on F. solani under Greenhouse Conditions

**Figure 6:**
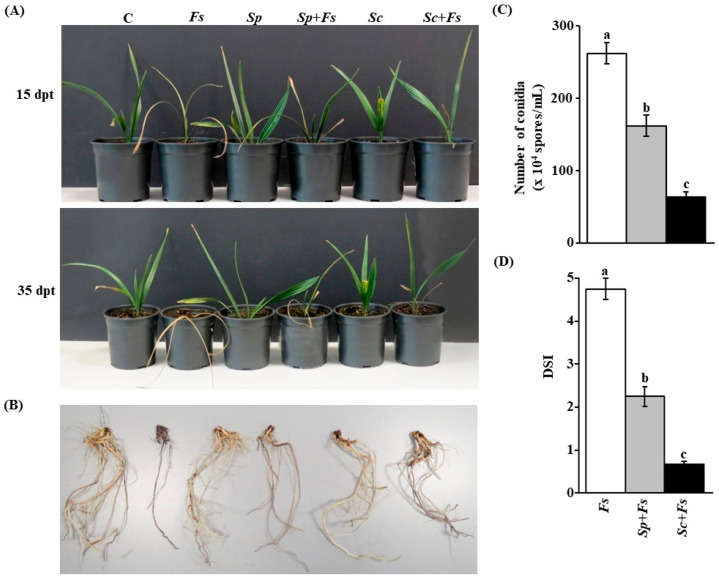
Preventive application of BCAs on *Fusarium solani*-inoculated date palm seedlings in the greenhouse. (**A**) Suppression of *Fusarium* wilt disease on date palm seedlings cv. Barhi using BCA1 *Streptomyces polychromogenes* UAE2 (isolate #16) and BCA2 *Streptomyces coeruleoprunus* UAE1 (isolate #28) at 15 (top) and 35 (bottom) dpt; (**B**) and recovery of root tissues infected with *F. solani* and previously inoculated with BCA at 35 dpt. (**C**) Number of conidia (×10^4^ spores/mL); and (**D**) disease severity index (DSI) after recovery of the pathogen from affected date palm root tissues (*n* = 16) treated with BCAs at 35 dpt. In (**A**–**D**), seedlings inoculated with *F. solani* at 5 days after BCA treatments. In (**C**,**D**), values (means ± SE of 16 replicates from two independent experiments) with different letters are significantly different from each other at *p* = 0.05. In (**D**), DSI was based on a scale of 5:0 = no infection, 1 = 1–10%, 2 = 11–25%, 3 = 26–50%, 4 = 51–75% and 5 = 76–100% damage including necrosis, white area in leaves or rotting in roots. Isolates #16 and #28 represent *Streptomyces polychromogenes* UAE2 (BCA1) and *Streptomyces coeruleoprunus* UAE1 (BCA2), respectively. BCA, biological control agent; C, non-inoculated seedling (control); *Fs*, infected seedlings with *F. solani* only; *Sp* or *Sc*, seedlings inoculated with only *S. polychromogenes* UAE2 or *S. coeruleoprunus* UAE1, respectively; *Sp* + *Fs or Sc* + *Fs*, seedlings inoculated with the individual BCA (either *S. polychromogenes* UAE2 or *S. coeruleoprunus* UAE1, respectively) 5 days prior to *F. solani* inoculation; dpt, days post treatment.

To evaluate the BCAs in suppressing growth of *F. solani*, an *in vivo* experiment was conducted in the greenhouse. Typical disease symptoms of SDS after 10 dpi with *F. solani* were observed when the pathogenicity tests were done on date palm seedlings (Figure 6A). The disease progressed with time and leaves of infected seedlings showed distinct dryness and bending at 30 dpi. On the other hand, no symptoms of SDS were noticed in non-inoculated seedlings (C) or inoculated plants with any of the BCAs alone (Figure 6A,B).

To assess the preventive effect of individual BCAs on infected seedlings with *F. solani*, we treated plants with either *S. polychromogenes* UAE2 (isolate #16, BCA1) or *S. coeruleoprunus* UAE1 (isolate #28, BCA2) at 5 days before inoculation with *F. solani*. When applied, individual treatments of the BCAs tested suppressed SDS to varying degrees. At 15 dpt, the inoculated plants treated with BCA1 (*Sp* + *Fs*) and BCA2 (*Sc* + *Fs*) started to recover, which was in contrast to *F. solani*-inoculated plants (*Fs*) without BCA treatments (Figure 6A,B). We also observed that newly fresh leaves emerged from the center of the crown area of date palm seedlings treated with any of the BCAs at 35 dpt (Figure 6A). This confirmed our *in vitro* results of the inhibitory effect of the BCAs on mycelial growth of *F. solani*. We also examined the root system of inoculated seedlings with either the pathogen or BCAs alone, and the combination application of *Sp* + *Fs* and *Sc* + *Fs*. At the end of the experiment, plants inoculated with the examined BCA candidates following inoculation with *F. solani* recovered when compared with seedlings inoculated with *F. solani* only, and appeared to be healthy and were, more or less, comparable to plants that were inoculated with its corresponding BCA treatments (Figure 6B). This suggests that both BCA candidates effectively inhibit *F. solani* growth *in vivo*.

In addition, we compared the responses of the pathogen to BCA treatments to determine their effects on numbers of conidia. Consequently, the conidia counts of *F. solani* recovered from the roots of treated date palm seedlings were determined. The BCA2 candidate (*Sc* + *Fs*) caused a greater reduction in the number of conidia than by BCA1 candidate (*Sp* + *Fs*; Figure 6C). Approximately 4- and 2.5-fold reduction in total conidia numbers of *F. solani* was observed in seedlings treated with BCA2 compared to that with the pathogen and BCA1 treatment, respectively. Thus, the pathogen was less aggressive to support the progression of SDS than any of the BCAs applied, while only a moderate inhibitory effect was observed in the case of BCA1 treatment.

To confirm these results, DSI was calculated in plants treated with *Sp* or *Sc* and inoculated with *F. solani*. We observed significant (*p* < 0.05) differences in DSI among treatments. It was not surprising that seedlings infected with *F. solani* progressed with SDS during the period of infection (Figure 6D). At 35 dpt, the results obtained in the preventive application of BCAs were clearly perceivable since both BCAs showed significant (*p* < 0.05) difference in DSI measurements compared to plants infected with *F. solani*. Thus, there was a dramatic decrease in DSI in *S. coeruleoprunus*-treated seedlings at 35 dpt, when compared with that of the same plants inoculated with the pathogen or treated with *S. polychromogenes* UAE2.

It is clear that the DSI of the application of *S. polychromogenes* UAE2 or *S. coeruleoprunus* UAE1 was significantly lower than of *F. solani*. This was evident when DSI dropped from 4.75 in *F. solani*-infected plants to 2.25 in *S. polychromogenes* and 0.67 in *S. coeruleoprunus* at 35 dpt, providing 53% and 86% reduction in disease development, respectively (Figure 6D). The preventive application of *S. coeruleoprunus* UAE1 at 5 days before inoculation with *F. solani* was more potent in suppressing the pathogen growth. Together, *S.* *coeruleoprunus* UAE1 (BCA2) possessing diffusible antifungal metabolites activities has stronger antifungal effects on *F. solani* than the CWDEs-producing isolate *S.* *polychromogenes* UAE2 (BCA1) under greenhouse conditions.

## 4. Discussion

Biotic stresses adversely affect growth, productivity, and trigger a series of morphological, physiological, biochemical, and molecular changes in plants [66,67,68]. Successful disease control requires thorough knowledge of the causal agent and symptoms associated with the disease. *F. solani* DSM-106836 is considered the major fungal pathogen causing SDS on date palm in the UAE [30]. The fungus grows into host tissues, eventually leading to complete dryness and death of palm trees. According to [29], *F. solani* is the most abundant fungal pathogen associated with date palm root diseases in the UAE. Thus far, chemical-based means is known for their curative properties to suppress the fungal pathogen infection on host plants. Previously, the fungicide Cidely^®^ Top (cyflufenamid and difenoconazole) was highly effective in inhibiting mycelial growth of several fungal pathogens [2,3,6], including *F. solani* on date palm [30]. The efficacy of chemical fungicides against the pathogen may last, however, for a short period of time, i.e., usually within a few days of infection. To develop environmentally-friendly solutions to combat SDS, we aimed to isolate endophytic actinobacteria and characterize their preventive antifungal activities against *F. solani* under greenhouse conditions.

For that reason, we obtained 31 endophytic actinobacteria isolates from within the root tissues of healthy date palm trees. In agreement with other studies, we found that *Streptomyces* was the most dominant genus that acts as reservoirs of novel bioactive secondary metabolites [23,69,70]. Other members of rare NSA genera were also reported [6], but none of these were able to manage SDS in this study (Table 1).

As suggested by [71], actinobacterial isolates were first identified according to their *in vitro* abilities to produce either CWDEs or diffusible antifungal metabolites against *F. solani*. Our data confirmed previous reports about the importance of actinobacteria as effective BCAs against phytopathogens [5,6,16,72]. To eliminate weak BCA isolates obtained from the primary *in vitro* screening assays, potential actinobacterial antagonists to *F. solani* were tested for a period of 12 weeks for their endophytic abilities to grow and increase in numbers in root tissues of date palm under greenhouse conditions. Such bioassays have been previously reported to measure the internal colonization of host tissues by endophytic isolates for effective active secondary metabolites [13,14], which ensures more protection in plants to subsequent infestation by the same (e.g., *F. solani*) or other phytopathogens. In alignment with that, our results successfully revealed one endophytic BCA candidate that was associated with the production of CWDEs (chitinase and ß-1,3-glucanase) and another linked with antibiosis, thus both were selected for further greenhouse experiments. Isolate #16 (BCA1) was characterized as the most promising CWDEs-producing strain, whereas isolate #28 (BCA2) was considered as a prominent strain due to the highly diffusible antifungal metabolites produced. Both isolates showed comparable *in vitro* production of other biochemical substances (e.g., volatile compounds and siderophores). According to the 16S rRNA sequence similarity with other *Streptomyces* spp., BCA1 and BCA2 were identified as *S. polychromogenes* UAE2 and *S. coeruleoprunus* UAE1, respectively.

Actinobacteria have long been known to suppress growth of phytopathogens and enhance plant resistance. Different modes of actions have been used by BCAs to control plant fungal diseases, including antibiosis, production of enzymes, enzyme inhibitors and signaling molecules, and/or immunomodulators [73]. In that sense, some microorganisms can parasitize on and kill fungi by secreting lytic enzymes such as chitinases, β-1,3-glucanases, proteases, and lipases [74]. Hence, antagonists synthesizing CWDEs can adversely affect mycelial growth and/or conidial walls of the fungal pathogen [75]. In the current study, one of the criteria for the potential BCA selection was based on the production of CWDEs (e.g., chitinase and ß-1,3-glucanase) against *F. solani*. Several SA, such as *Streptomyces viridicans*, *Streptomyces viridodiasticus*, and *Streptomyces* spp., have been tested previously for their abilities to produce chitinases against phytopathogens [38,52,76]. Moreover, ß-glucanase-producing SA isolates have been reported to hydrolyze fungal cell-wall glucans, resulting in the suppression of root rot of raspberry caused by *Phytophthora fragariae* var. *rubi* [37] and *Fusarium* wilt of cucumber [52]. The actinobacterial isolates, *Micromonospora tulbaghiae* UAE1, and *Streptomyces coelicoflavus* UAE1, have been reported to secret CWDEs against fungal pathogens on mango and royal poinciana, respectively [6,72]. Five *Streptomyces* spp. have served as BCAs producing chitinases against *Fusarium* wilt (bayoud) disease of date palm caused by *Fusarium oxysporum* f.sp. *albedinis* [77].

Antibiosis is another major mechanism of which a particular BCA can secrete metabolites as a source of biofungicide to mimic the development, survival, dispersal, and reproduction of plant pathogens. Several studies have reported actinobacterial BCAs, mainly *Streptomyces* spp., employing antibiosis to protect plants against diseases caused by fungal pathogens. Such examples may include *Streptomyces lydicus* WYEC108 against fungal root and seed rots [78], *Streptomyces janthimts* and *Streptomyces cinerochromogenes* against cavity-spot disease of carrots [17], *Streptomyces globosus* UAE1 against black scorch disease on date palm [5], and *Streptomyces cavourensis* UAE1 against mango dieback disease [6]. In addition, *Streptomyces violaceusniger* G10 has served as a BCA possessing antibiosis against wilt disease of banana caused by *Fusarium oxysporum* f.sp. *cubense* [79]. Although *Streptomyces rochei* UAE2 isolated from rhizosphere soils of royal poinciana confirms our results that antibiosis is an effective mechanism for BCAs, *Streptomyces antibioticus* UAE1 was relatively superior against the causal agent stem canker disease, *Neoscytalidium* *dimidiatum* [72]. This can be attributed to the multiple mode of actions in *S. antibioticus* UAE1 to produce synergistic effects against the fungus.

The use of a single or a combination of BCA(s) with multiple modes of action is expected to be a better method for developing biocontrol positive effects [80]. For instance, a mixture of three endophytic actinobacteria, namely *Actinoplanes campanulatus*, *Micromonospora chalcea*, and *Streptomyces spiralis*, possessing different modes of action has been investigated for better efficacy against *Pythium aphanidermatum* and prevention of root rot and crown rot diseases of cucumber than an individual application of *S. spiralis* [12]. These three antagonists produced high levels of ß-1,3, ß-1,4, and ß-1,6 glucanases. In addition to their ability to produce CWDEs, *M. chalcea* and *S. spiralis* can produce volatile inhibitory compounds, whereas *A. campanulatus* and *S. spiralis* can produce diffusible antifungal metabolites [12]. Mango plants pre-inoculated with *Streptomyces samsunensis* UAE1 showing multiple biocontrol mechanisms resulted in higher levels of disease protection against *Lasiodiplodia theobromae* than those pre-inoculated with the individual isolates of *Streptomyces cavourensis* or *M. tulbaghiae* showing one mode of action [6].

The rhizospheric isolate, *S. antibioticus* UAE1, possessing synergistic mechanisms of antagonism (production of CWDEs and antibiosis), had additive effect of suppression to stem canker disease [72]. Although the actinobacterial isolates, *Streptomyces* *wuyuanensis* UAE1, and *Streptomyces* *griseorubens* UAE2 have the ability to produce CWDEs and siderophores, as well as antibiosis and enhance royal poinciana resistance against *N. dimidiatum*, the latter isolate had augmentative influence on suppressing the symptoms associated with stem canker disease [16]. This is because of the production of high levels of ACC deaminase by *S. griseorubens* that was absent in the other BCA.

There are many factors/preferences for choosing one or multiple mode(s) of action for an envisaged application of a single or a combination of BCA(s) to protect plants [71]. Some factors such as the characteristics of the BCA(s), the nature of the mode(s) of action, the risks to the humans or environment, resistance development against the BCA by the pathogen, the pathogen specificity, and its dependency on environmental conditions and crop physiology can have an impact on the selection of new antagonists. Along with that, the actinobacteria *S. polychromogenes* UAE2 and *S. coeruleoprunus* UAE1, reported in the current study, had antifungal effects and suppressed SDS. This was evident when any of the examined BCAs was applied prior to *F. solani* inoculation. Our results showed that there was significant (*p* < 0.05) reduction in the DSI in inoculated date palm seedlings and the conidial counts of *F. solani* (Figure 6), in addition to the malformation of the pathogen’s internal structures (Figure 4). This indicates that both isolates showed effective biocontrol activities against *F. solani*, albeit their mode of action. We noticed that *S. coeruleoprunus* UAE1 displayed increased resistance to the pathogen, considering this treatment as an effective biofungicide on date palm seedlings to control *F. solani*. This could be attributed to the establishment of total biomass of the BCA population needed to provide systemic suppression of *F. solani* without damaging the host plant.

The development of stable populations of BCAs adapted to harsh environment plays a key role to increase the productivity of commercial BCA products [70,81]. The tested actinobacterial BCAs—in our study—were capable to tolerate extreme hot temperatures and to grow in arid environments as reported by [82]. We argue that the use of the BCAs identified in this study to control SDS on date palm can be enhanced by their use in a consortium. Furthermore, mixtures with additives or chemical fungicides (at very low concentration) can be integrated in IDM programs as eco-friendly practices [83,84]. Field-testing of *S. polychromogenes* UAE2 and *S. coeruleoprunus* UAE1 is on top of our priorities to determine the effectiveness against *F. solani*.

## Figures and Tables

**Table 1:** Antagonism against *Fusarium solani* by endophytic actinobacterial isolates obtained from within date palm root tissues.

Species	Isolate	Clearing Zone Diameter ^a^	Inhibition Zone Diameter ^b^	Production of		
		(mm)		VC ^c^	Siderophore ^d^	HCN ^d^
**SA**						
*Streptomyces*	#1	0.0	34.3 ± 0.7 *ab*	+	-	-
	#5	32.2 ± 1.0 *a*	0.0	+	+	-
	#7	0.0	41.6 ± 1.7 *c*	-	+	-
	#9 ^e^	0.0	0.0	-	-	-
	#13	41.4 ± 2.1 *b*	0.0	-	+	-
	#15	0.00	37.3 ± 0.8 *b*	+	-	-
	#16 ^e^	55.4 ± 2.2 *c*	0.0	+	+	-
	#22	0.0	47.3 ± 1.2 *d*	+	+	-
	#24	37.1 ± 1.1 *ab*	0.0	-	-	-
	#25	0.0	31.4 ± 0.4 *a*	-	+	-
	#28 ^e^	0.0	48.2 ± 2.0 *d*	+	+	-
	#31	42.7 ± 1.3 *b*	0.0	-	+	-
**NSA**						
*Actinoplanes*	#3	0.0	32.8 ± 0.9 *a*	+	-	-
*Dactylosporangium*	#8	0.0	42.2 ± 1.6 *c*	-	+	-
*Microbispora*	#21	33.8 ± 2.0 *a*	0.0	+	-	-
*Micromonospora*	#29	0.0	47.8 ± 1.1 *d*	+	+	-
	#17	54.6 ± 1.9 *c*	0.0	+	+	-

^a^ Production of CWDEs on mycelial fragment agar. ^b^ Production of diffusible antifungal metabolites against *F. solani* using the cut-plug method. ^c^ +, fungicidal effect; -, no fungicidal effect. ^d^ +, produced; -, not produced. ^e^ Isolate #9 is a non-antifungal- and non-CWDE-producing positive control. Isolates #16 and #28 represent *Streptomyces polychromogenes* UAE2 (BCA1) and *Streptomyces coeruleoprunus* UAE1 (BCA2), respectively. Values are means ± SE of six replicates for *in vitro* experiments for production of CWDEs and diffusible antifungal metabolites. Values within each column, followed by the same letter, are not significantly (*p* > 0.05) different according to Duncan’s multiple range test. VC, volatile antifungal compounds; BCA, biological control agent; SA, streptomycete actinobacteria; NSA, non-streptomycete actinobacteria; CWDEs, cell-wall-degrading enzymes.

**Table 2:** Effects of the crude culture filtrate of the two BCA candidates obtained from FMEB and CCB on the mycelial, conidial, and germ tube characteristics of *Fusarium solani*.

Media	Isolate	Culture Filtrate (%)	Colony Diameter (mm)	Mycelial Dry Weight (g)	Conidia Germination (%)	Germ Tube Length (µm)
CCB	#16	0	97.7 ± 0.4 *d*	76.4 ± 2.8 *d*	87.8 ± 0.5 *d*	63.4 ± 1.2 *d*
		25	56.3 ± 4.2 *c*	45.2 ± 2.2 *c*	55.1 ± 5.3 *c*	33.3 ± 4.1 *c*
		50	15.5 ± 2.8 *b*	13.8 ± 1.1 *b*	17.6 ± 2.4 *b*	7.9 ± 1.0 *b*
		100	0.0 *a*	0.0 *a*	1.0 ± 0.2 *a*	0.0 *a*
FMEB	#28	0	98.0 ± 0.6 *d*	74.5 ± 1.4 *d*	86.6 ± 1.0 *d*	50.2 ± 1.7 *d*
		25	61.9 ± 3.8 *c*	39.9 ± 4.4 *c*	51.7 ± 3.5 *c*	25.7 ± 5.7 *c*
		50	13.2 ± 1.6 *b*	7.8 ± 1.3 *b*	14.3 ± 2.2 *b*	11.2 ± 3.2 *b*
		100	0.0 *a*	0.0 *a*	0.9 ± 0.3 *a*	1.9 ± 0.4 *a*

Values are means ± SE of six replicates. Values with the same letter within a column for each BCA are not significantly (*p* > 0.05) different, according to Duncan’s multiple range test. Isolates #16 and #28 represent *Streptomyces polychromogenes* UAE2 (BCA1) and *Streptomyces coeruleoprunus* UAE1 (BCA2), respectively. BCA, biological control agent; CCB, colloidal chitin broth; FMEB, fish meal extract broth.

## Data Availability

All sequences were deposited in Genbank (NCBI; https://www.ncbi.nlm.nih.gov/ (accessed on 17 December 2021)) with accession numbers OK560620 for *Streptomyces polychromogenes* UAE2 and OK560621 for *Streptomyces* *coeruleoprunus* UAE1.

## Supplementary Materials

The following are available online at https://www.mdpi.com/article/10.3390/jof8010008/s1, Figure S1: Colonies of actinobacteria isolated from within surface-sterilized date palm roots grown on inorganic salt starch agar plates. White and red arrows represent the streptomycete and non-streptomycete (*Actinoplanes, Dactylosporangium*, *Micromonospora* and *Microbispora* spp.) actinobacterial colonies, respectively, Figure S2: Production of CWDEs and diffusible antifungal metabolites by BCAs active against *Fusarium solani*. Production of (A) chitinase enzymes; and (B) CWDEs by isolate #16 and not #28. Inhibition of *F. solani* mycelial growth by isolate #28 and not #16 using (C) cut-plug method; and (D) cup plate method. In (A), production of chitinase by isolate #16 compared to the non-chitinase producing isolate #28 on CCA. In (B), production of CWDEs by isolate #16 compared to the non-chitinase producing isolate #28 on MFA. In (C), plugs of isolate #16 and #28 grown on FMEA plates were cut and placed on PDA plates seeded with *F. solani*. In (D), wells in PDA plates seeded with *F. solani* were inoculated with filter-sterilized crude culture filtrates of isolates #16 or #28 grown on FMEA. In (C,D), the diffusible antifungal metabolite-producing isolate #28 compared to the non-diffusible antifungal metabolite-producing isolate #16. Isolates #16 and #28 represent *Streptomyces polychromogenes* UAE2 (BCA1) and *Streptomyces coeruleoprunus* UAE1 (BCA2), respectively. BCA, biological control agent; CWDEs, cell wall-degrading enzymes; CCA, colloidal chitin agar; MFA, mycelial fragment agar; FMEA, fish meal extract agar; PDA, potato dextrose agar, Figure S3: Effect of the BCAs on mycelial growth of *Fusarium solani* and production of siderophores by the BCAs. Dialysis membrane overlay technique using (A) CCA; or (B) FMEA plates; and (C) production of siderophores by selected actinobacterial strains isolated from healthy date palm tissues. In (A), inhibition of *F. solani* mycelial growth was only noticed in the chitinase-producing isolate #16 compared to the chitinase non-producing isolate #28. In (B), inhibition of *F. solani* mycelial growth was observed only by the diffusible antifungal metabolite-producing isolate #28 compared to the non-diffusible antifungal metabolite-producing isolate #16. In (C), isolates were tested on chrome azurol S agar plates; and yellow halo surrounding the colony indicates the excretion of siderophores. Isolates #16 and #28 were considered as siderophore-producing isolates. The non-siderophore-producing actinobacterial strain #9 was used as a control isolate. Isolates #16 and #28 represent *Streptomyces polychromogenes* UAE2 (BCA1) and *Streptomyces coeruleoprunus* UAE1 (BCA2), respectively. BCA, biological control agent; CWDEs, cell wall-degrading enzymes; FMEA, fish meal extract agar; CCA, colloidal chitin agar, Table S1: *In vitro* comparisons of antagonistic activities between the potential CWDEs-producing isolate #16 (BCA1) and the antifungal metabolite-producing isolate #28 (BCA2) against *Fusarium solani*, Figure S4: Molecular, cultural and morphological characteristics of isoalte #16, *Streptomyces polychromogenes* UAE2. Identification of *S. polychromogenes* UAE2 (BCA1) according to (A) the dendrogram that showing the phylogenetic relationships between *S. polychromogenes* UAE2 (NCBI accession #: OK560620) and other members of *Streptomyces* spp. based on the 16S rRNA sequences prepared by the neighbor-joining method; (B) grayish pink aerial mycelium (left) and yellowish brown substrate mycelium (right) growing on (ISP medium 3) supplemented with yeast extract, and (C) scanning electron micrograph (4300X) of the long, straight spore chains (section rectiflexibiles) and smooth-surfaced spores of *S. polychromogenes* UAE2. In (A), numbers at nodes indicate percentage levels of bootstrap support based on a neighbor-joining analysis of 500 resampled datasets. GenBank accession numbers are given in parentheses. BCA, biological control agent.
