# Supplementary material for: Biocontrol Potential of Endophytic Actinobacteria against Fusarium solani, the Causal Agent of Sudden Decline Syndrome on Date Palm in the UAE
Source: J Fungi (Basel). 2021 Dec 23;8(1):8. doi: 10.3390/jof8010008 (PMC8779766; doi:10.3390/jof8010008)
Supplement: Supplementary file 1 [file jof-08-00008-s001.zip › jof-1511833-supplementary.pdf]

Supplementary Material

Biocontrol Potential of Endophytic Actinobacteria against *Fusarium solani*, the Causal Agent of Sudden Decline Syndrome on Date Palm in the UAE

Aisha A. Alblooshi, Gouthaman P. Purayil, Esam Eldin Saeed, Gaber A. Ramadan,

Saeed Tariq, Amna S. Altaee, Khaled A. El-Tarabily *, and Synan F. AbuQamar *

* Correspondence: ktarabily@uaeu.ac.ae (K.A.E.-T.)
sabuqamar@uaeu.ac.ae (S.F.A.)

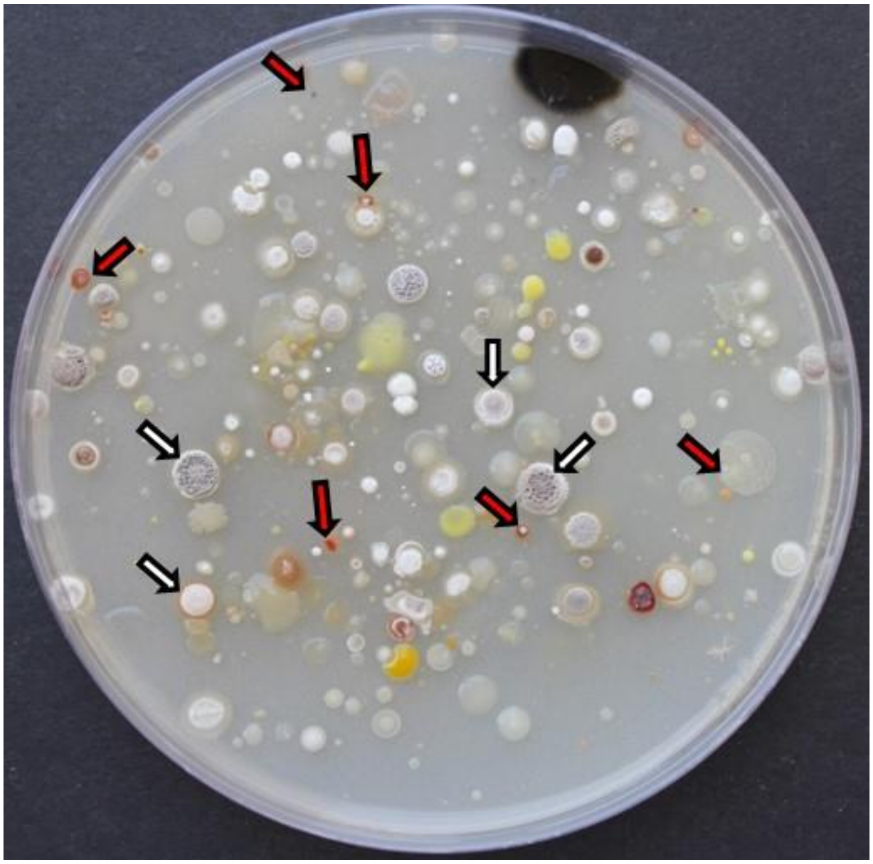

Figure S1. Colonies of actinobacteria isolated from within surface-sterilized date palm roots grown on inorganic salt starch agar plates. White and red arrows represent the streptomycete and non-streptomycete (*Actinoplanes, Dactylosporangium*, *Micromonospora* and *Microbispora* spp.) actinobacterial colonies, respectively.

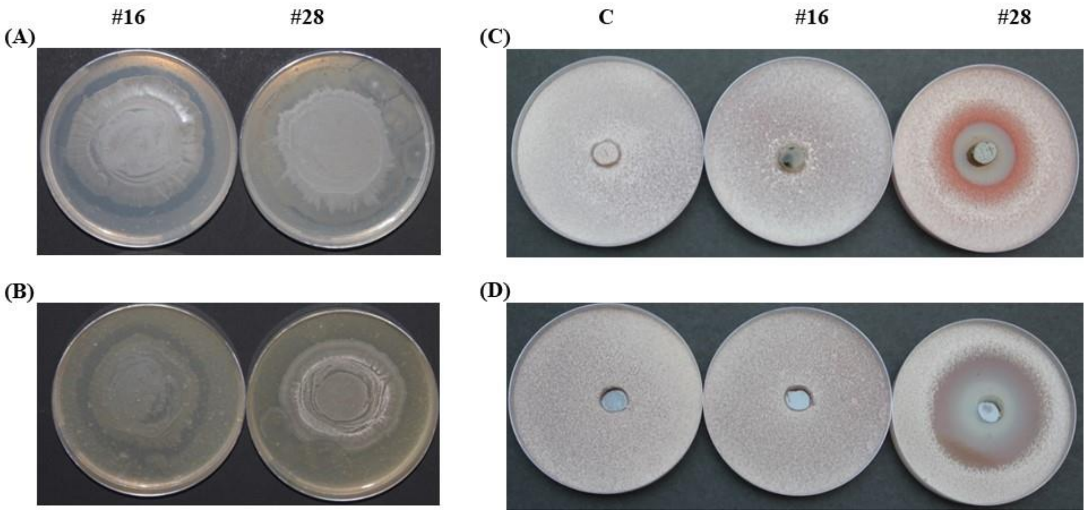

Figure S2. Production of CWDEs and diffusible antifungal metabolites by BCAs active against *Fusarium solani*. Production of (**A**) chitinase enzymes; and (**B**) CWDEs by isolate #16 and not #28. Inhibition of *F. solani* mycelial growth by isolate #28 and not #16 using (**C**) cut-plug method; and (**D**) cup plate method. In (**A**), production of chitinase by isolate #16 compared to the non-chitinase producing isolate #28 on CCA. In (**B**), production of CWDEs by isolate #16 compared to the non-chitinase producing isolate #28 on MFA. In (**C**), plugs of isolate #16 and #28 grown on FMEA plates were cut and placed on PDA plates seeded with *F. solani*. In (**D**), wells in PDA plates seeded with *F. solani* were inoculated with filter-sterilized crude culture filtrates of isolates #16 or #28 grown on FMEA. In (**C & D**), the diffusible antifungal metabolite-producing isolate #28 compared to the non-diffusible antifungal metabolite-producing isolate #16. Isolates #16 and #28 represent *Streptomyces polychromogenes* UAE2 (BCA1) and *Streptomyces coeruleoprunus* UAE1 (BCA2), respectively. BCA, biological control agent; CWDEs, cell wall-degrading enzymes; CCA, colloidal chitin agar; MFA, mycelial fragment agar; FMEA, fish meal extract agar; PDA, potato dextrose agar.

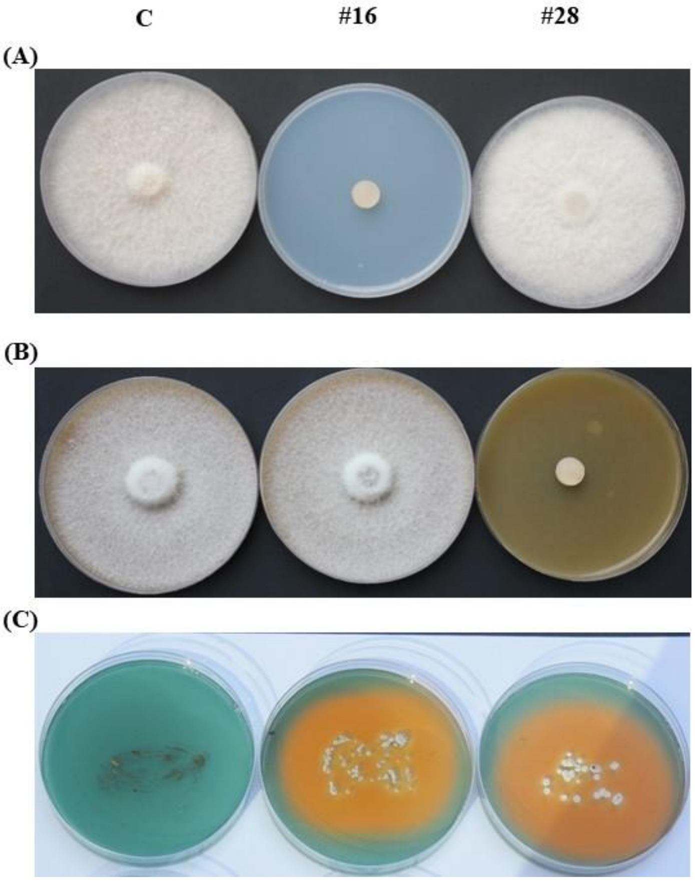

Figure S3. Effect of the BCAs on mycelial growth of *Fusarium solani* and production of siderophores by the BCAs. Dialysis membrane overlay technique using (A) CCA; or (B) FMEA plates; and (C) production of siderophores by selected actinobacterial strains isolated from healthy date palm tissues. In (A), inhibition of *F. solani* mycelial growth was only noticed in the chitinase-producing isolate #16 compared to the chitinase non-producing isolate #28. In (B), inhibition of *F. solani* mycelial growth was observed only by the diffusible antifungal metabolite-producing isolate #28 compared to the non-diffusible antifungal metabolite-producing isolate #16. In (C), isolates were tested on chrome azurol S agar plates; and yellow halo surrounding the colony indicates the excretion of siderophores. Isolates #16 and #28 were considered as siderophore-producing isolates. The non-siderophore-producing actinobacterial strain #9 was used as a control isolate. Isolates #16 and #28 represent *Streptomyces polychromogenes* UAE2 (BCA1) and *Streptomyces coeruleoprunus* UAE1 (BCA2), respectively. BCA, biological control agent; CWDEs, cell wall-degrading enzymes; FMEA, fish meal extract agar; CCA, colloidal chitin agar.

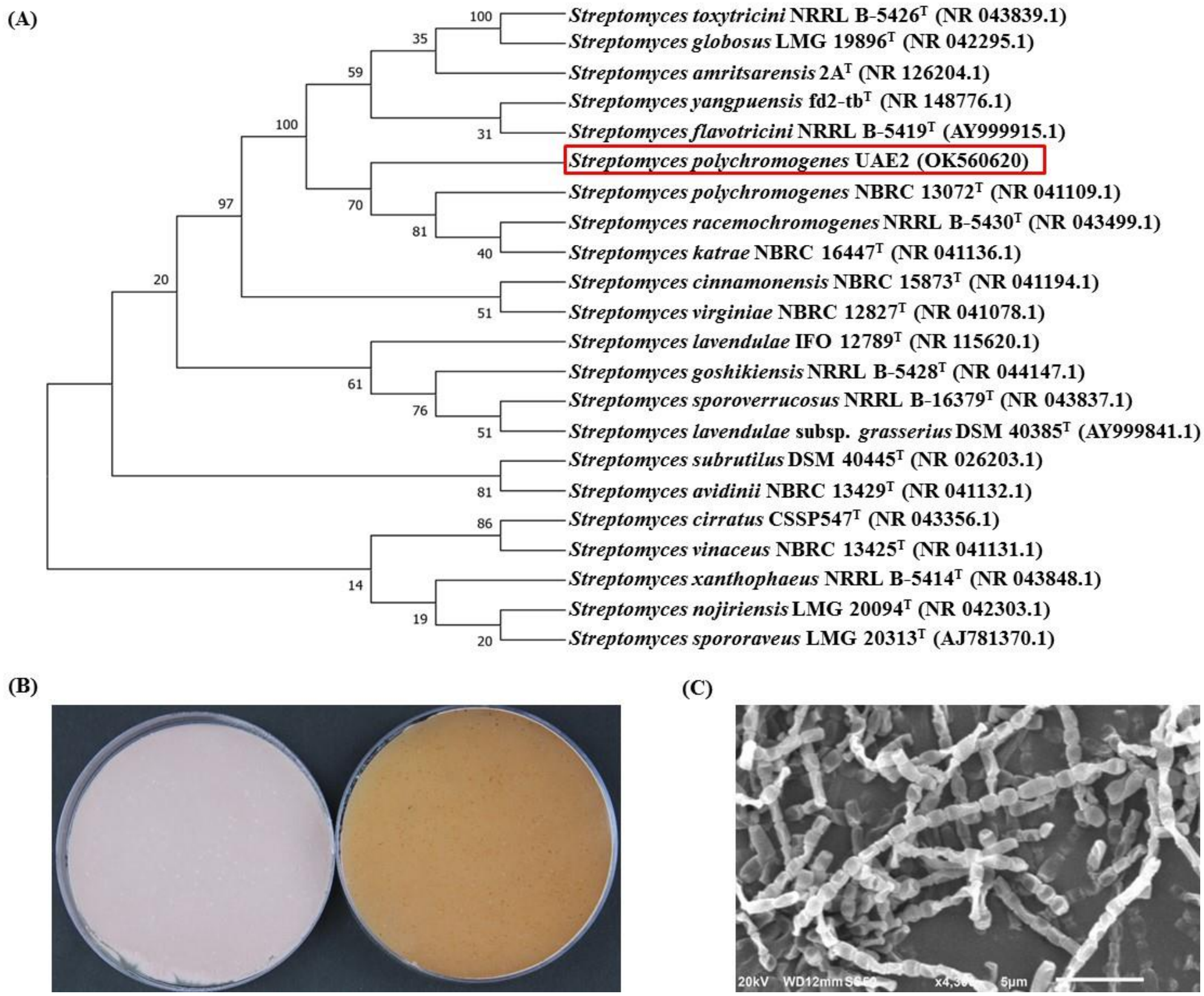

Figure S4. Molecular, cultural and morphological characteristics of isoalte # 16, *Streptomyces polychromogenes* UAE2. Identification of *S. polychromogenes* UAE2 (BCA1) according to (A) the dendrogram that showing the phylogenetic relationships between *S. polychromogenes* UAE2 (NCBI accession #: OK560620) and other members of *Streptomyces* spp. based on the 16S rRNA sequences prepared by the neighbor-joining method; (B) grayish pink aerial mycelium (left) and yellowish brown substrate mycelium (right) growing on (ISP medium 3) supplemented with yeast extract, and (C) scanning electron micrograph (4,300X) of the long, straight spore chains (section rectiflexibiles) and smooth-surfaced spores of *S. polychromogenes* UAE2. In (A), numbers at nodes indicate percentage levels of bootstrap support based on a neighbor-joining analysis of 500 resampled datasets. GenBank accession numbers are given in parentheses. BCA, biological control agent.

Table S1. *In vitro* comparisons of antagonistic activities between the potential CWDEs-producing isolate #16 (BCA1) and the antifungal metabolite-producing isolate #28 (BCA2) against *Fusarium solani*.

Activities	Isolate	
	**#16**	**#28**
**Production of diffusible metabolites using**		
cup plate (diameter of inhibition zone in mm)	0.0 *a*	58.35±3.1 *b*
dialysis membrane from fish meal extract agar plates [a]	-	+
**Production of chitinase using** dialysis membrane from colloidal chitin agar plates [a]	+	-
**Chitinase from**		
colloidal chitin (U ml^{-1}) [b]	9.66±1.46 *b*	0.0 *a*
N. dimidiatum cell-wall (U ml^{-1}) [b]	3.96±1.02 *b*	0.0 *a*
**ß-1,3-glucanase from**		
laminarin (U ml^{-1}) [c]	7.58±1.30 *b*	0.0 *a*
N. dimidiatum cell-wall (U ml^{-1}) [c]	1.54±0.74 *b*	0.0 *a*

[a] + = fungicidal effect; - = no fungicidal effect.
[b] A unit of chitinase was expressed as the amount of the enzyme that released 1 μmol of N-acetyl-D-glucosamine mg^{-1} protein h^{-1}. This indicates that isolate #16 (BCA1) produced high levels of chitinase.
[c] A unit of ß-1,3-glucanase was expressed as the amount of the enzyme that released 1 μmol of glucose mg^{-1} protein h^{-1}. This indicates that isolate #16 (BCA1) produced high levels of ß-1,3-glucanase.
Values are means±SE from six replicates. Values with the same letter within a row are not significantly (P>0.05) different according to Duncan's multiple range test.
Isolates #16 and #28 represent *Streptomyces polychromogenes* UAE2 (BCA1) and *Streptomyces coeruleoprunus* UAE1 (BCA2), respectively.
BCA, biological control agent.
